# *Plasmodium cynomolgi* Infections Not Found in Microscopy-Diagnosed Malaria Cases across Sabah, Malaysia

**DOI:** 10.4269/ajtmh.24-0264

**Published:** 2024-11-12

**Authors:** Lydia S. Murdiyarso, Giri S. Rajahram, Angelica F. Tan, Kim A. Piera, Timothy William, Damian A. Oyong, Sitti Saimah Binti Sakam, Jenarun Jelip, Jiloris Dony, Anisah Jantim, Roddy Teo, Abdul Marsudi Manah, Bridget E. Barber, Nicholas M. Anstey, Matthew J. Grigg

**Affiliations:** ^1^ Global and Tropical Health Division, Menzies School of Health Research, Charles Darwin University, Darwin, Australia;; ^2^ Infectious Disease Society Kota Kinabalu Sabah – Menzies School of Health Research Clinical Research Unit, Kota Kinabalu, Malaysia;; ^3^ Clinical Research Centre, Queen Elizabeth Hospital, Ministry of Health, Kota Kinabalu, Malaysia;; ^4^ Queen Elizabeth Hospital II, Kota Kinabalu, Malaysia;; ^5^ Burnet Institute, Melbourne, Australia;; ^6^ Vector Borne Disease Sector, Ministry of Health, Putrajaya, Malaysia;; ^7^ Kota Kinabalu Public Health Laboratory, Ministry of Health, Kota Kinabalu, Malaysia;; ^8^ Public Health Research Section, Sabah State Department of Health, Kota Kinabalu, Malaysia;; ^9^ Vector Borne Disease Unit, Sabah State Department of Health, Kota Kinabalu, Malaysia;; ^10^ QIMR Berghofer Medical Research Institute, Brisbane, Australia

## Abstract

Zoonotic malaria presents a major public health challenge in Southeast Asia. *Plasmodium cynomolgi* coinfects the same macaque hosts and mosquito vectors as the most common cause of zoonotic malaria, *Plasmodium knowlesi*. *Plasmodium cynomolgi* appears morphologically similar to *Plasmodium vivax* on microscopy and can amplify *P. vivax* polymerase chain reaction (PCR) assays, confounding transmission estimates. We screened 2,103 samples for *P. cynomolgi* across all 26 districts in Sabah, Malaysia, from 2010 to 2021. Samples comprised 1,425 *P. knowlesi*, 256 *P. vivax*, 293 *P. falciparum,* and 31 *Plasmodium malariae* PCR-confirmed malaria cases and 100 malaria microscopy-positive and species-specific PCR-negative samples. A nested PCR assay targeting *P. cynomolgi*-specific 18S small subunit ribosomal ribonucleic acid with a detection limit of ∼2 parasites/*µ*L was conducted on whole blood samples. No *P. cynomolgi* infections were detected. Symptomatic *P. cynomolgi* co-infections appear rare in Malaysia, although prevalence may be underestimated owing to the absence of routine molecular screening and the sensitivity of available assays.

Zoonotic malaria remains a public health concern, posing a major threat to malaria elimination in Southeast Asia.^1^ In Malaysia, where human malaria transmission has been steadily controlled through long-term public health efforts to the point of elimination, the prevalence of zoonotic malaria has increased.^2^^,^^3^ Although *Plasmodium knowlesi* causes most reported cases of zoonotic malaria, several cases of zoonotic *P. cynomolgi* infection have been reported in both Peninsular and East Malaysia,^4^^–^^6^ Thailand, and Cambodia.^7^^–^^9^ Interestingly, *P. cynomolgi* human infections are commonly reported as co-infections with *P. knowlesi*.^6^^–^^8^

*Plasmodium cynomolgi* and *P. knowlesi* share natural monkey hosts, including the long-tailed (*Macaca fascicularis)* and pig-tailed (*Macaca nemestrina*) macaques,^10^ which share an overlapping geographic distribution across large areas of Southeast Asia.^10^ *Plasmodium cynomolgi* and *P. knowlesi* co-infections are common in macaques and mosquitos.^11^^–^^13^ Importantly, within *M. fascicularis* populations in Southeast Asia, *P. cynomolgi* is more prevalent and widely distributed than any other macaque *Plasmodium* species (*Plasmodium inui, Plasmodium fieldi,* and *Plasmodium coatneyi*), albeit with some geographical heterogeneity in *Plasmodium* species composition.^14^

Morphologically, *P. cynomolgi* asexual stages are indistinguishable from those of the human parasite *P. vivax* under standard light microscopy diagnosis.^10^ In addition, both species are genetically similar, with *P. cynomolgi* DNA cross-reacting with the primers in the commonly used *P. vivax*–specific nested 18S small subunit ribosomal ribonucleic acid (ssu rRNA) assay, although *P. cynomolgi*–specific primers do not amplify *P. vivax*.^4^^,^^8^ Like *P. vivax*, human infection with *P. cynomolgi* could also result in the formation of the dormant hypnozoite liver stage that causes relapses seen in natural macaque hosts.^10^ Owing to a limited number of studies and technical diagnostic constraints, even with molecular tools, it is unclear if the occurrence and potential public health threat of zoonotic *P. cynomolgi* is largely underreported.

In this study, we aimed to detect *P. cynomolgi* infections retrospectively from patients with symptomatic *Plasmodium* species infections enrolled in previous studies in Sabah, Malaysia.

To increase the likelihood of detecting *P. cynomolgi*, we selected available samples from a geographically representative subset of patients with uncomplicated malaria from ongoing hospital-based malaria surveillance studies in Sabah, Malaysia.^15^^–^^17^ *Plasmodium* species–specific PCR assays on these samples had previously been conducted for *P. knowlesi*, *P. vivax*, *P. falciparum*, *P. malariae*, and *Plasmodium ovale* spp. and reported as part of mandatory state-reference laboratory testing on all microscopy-positive case notifications,^18^ with an initial molecular screening step for *Plasmodium* genus not conducted.

Samples included 1) those that were PCR positive for single *P. knowlesi*, *P. vivax, P. falciparum*, *or P. malariae* infections and 2) those that were positive for malaria by routine hospital-based microscopy but negative by *Plasmodium* species–specific PCR (assumed false-positive for notification purposes).

Whole blood samples were collected in Ethylenediaminetetraacetic acid tubes and kept cool until stored at −80°C. To accurately identify *Plasmodium* species, genomic DNA was extracted from thawed whole blood using QIAamp 96 DNA Mini Blood Kit (Qiagen, Hilden, Germany) according to the manufacturer’s instructions. Extracted DNA of confirmed malaria cases with previously conducted PCR^19^^,^^20^ was amplified using a nested PCR assay targeting the 18S ssu rRNA gene, with the nest 1 targeting the *Plasmodium* genus (rPLU1 and rPLU5 primer), followed by a *P. cynomolgi* species–specific (CY2F and CY4R primer) nest 2, with an expected limit of detection of approximately 2 parasites/*µ*L.^21^ (Table 1).

**Table 1:** *Plasmodium cynomolgi–*specific primers

PCR	Primers	Primer sequence (5′–3′)	Annealing Temp	Reference
Nest 1	rPLU1	TCAAAGATTAAGCCATGCAAGTGA	58°C	Snounou and Singh^22^
	rPLU5	CCTGTTGTTGCCTTAAACTTC	–	–
Nest 2	CY2F	GATTTGCTAAATTGCGGTCG	66°C	Lee et al.^12^
	CY4R	CGGTATGATAAGCCAGGGAAGT	–	–

PCR = polymerase chain reaction.

Nested PCR amplifications were performed for 35 cycles in both rounds with the following cycling conditions: 4 minutes at 94°C for initial denaturation, 30 seconds at 94°C for denaturation, 1 minute at 58°C and 66°C for first and second PCR rounds, respectively, for annealing, and 2 minutes and 1 minute at 72°C for first and second PCR rounds, respectively, for extension, followed by 4 minutes at 72°C for the final extension. Positive *P. cynomolgi* control amplification was confirmed using 1.5% agarose gel electrophoresis with SYBR^™^ Safe (Invitrogen, Carlsbad, CA) and viewed using a Molecular Imager^®^ Gel Doc^®^ XR system (Bio-Rad, Hercules, CA). The donated positive *P. cynomolgi* DNA control included in the assay was derived from an infected macaque source.

There were 2,103 malaria samples included for *P. cynomolgi* co-infection screening from across all 26 administrative districts in Sabah and the island federal territory of Labuan, in addition to two patients from neighboring Lawas District in Sarawak, Malaysia, from 2010 to 2021. The median number of microscopy-diagnosed malaria cases included from each year was 174 (interquartile range [IQR]: 94–236). The highest number of samples was from the districts of Kota Marudu (17%) and Kudat (16%), followed by Pitas (12%) and Keningau (8%). The median number of available malaria samples from each district was 31 (IQR: 7–93), with eight districts contributing fewer than 10 samples each.

The screened samples comprised 1,425 (68%) *P. knowlesi* infections, followed by 293 (14%) *P. falciparum,* 256 (12%) *P. vivax*, 31 (1%) *P. malariae*, and 100 (5%) microscopy-positive and PCR species–specific negative (Table 2). *Plasmodium cynomolgi* was not detected in any of the samples tested. Both positive and negative controls for *P. cynomolgi* demonstrated appropriate results on gel electrophoresis, supporting reliable conduct of the assay.

**Table 2:** Demographic and clinical details

Variable	*Plasmodium* Species–Specific PCR Result					
	*Plasmodium knowlesi*	*Plasmodium vivax*	*Plasmodium falciparum*	*Plasmodium malariae*	Negative*	Total
Number Tested, *N* (%)	1,425 (68)	254 (12)	293 (14)	31 (1)	100 (5)	2,103 (100)
Male Sex, *N* (%)	1,159 (81)	185 (73)	223 (76)	23 (74)	75 (71)	1,660 (79)
Age, Median Years, (IQR) [range]	35 (24–49) [2–98]	24 (14–35) [1–79]	27 (17–40) [3–78]	21 (12–28) [5–60]	25 (16–44) [1–86]	32 (21–47) [1–98]
Parasite Count/*µ*L, Geometric Mean (95% CI), [range]	1,160 (1,033–1,302) [≤40–792,567]	3,265 (2,762–3,869) [46–84,403]	8,087 (6,402–10,214) [≤40–959,383]	1,324 (753–2,328) [71–12,615]	623 (491–789) [50–22,222]	–

IQR = interquartile range; PCR = polymerase chain reaction.

* Negative for species-specific PCR, positive on routine microscopy.

In this zoonotic malaria surveillance study, no *P. cynomolgi* infections were detected in previously confirmed *Plasmodium* species infections or in those negative on species-specific PCR with likely false-positive microscopy results. *Plasmodium cynomolgi* infections were not seen despite a large number of geographically diverse samples screened, a range of both low and high parasite counts within each *Plasmodium* species group, and the addition of a high-risk group of documented microscopy-positive patients with febrile illness negative on species-specific malaria PCR assays. Positive and negative controls worked well, suggesting that the assay used was robust and supported the validity of these findings to the threshold for detection of the assay.

Zoonotic infection with *P. cynomolgi* is considered a potential public health risk because of the wide distribution of natural macaque hosts with a parasite reservoir maintained by a dormant hypnozoite stage. Although human cases of *P. cynomolgi* infection have been described,^4^^–^^6^ including in two asymptomatic individuals from 867 samples from a cross-sectional survey conducted in northern Sabah,^5^ our study did not find evidence of *P. cynomolgi* infection from a broader geographical area in symptomatic microscopy-positive patients in Sabah.

Several reasons could be attributed to the lack of *P. cynomolgi* infections found in this study. The prevalence of zoonotic *P. cynomolgi* infection could be considerably lower than our sample size was powered to accurately detect. Reported human *P. cynomolgi* infections are undoubtedly of substantially lower prevalence compared with that of *P. knowlesi* infection in Sabah.^5^^,^^6^ Our findings are consistent with an absence of *P. cynomolgi* infections in a large longitudinal surveillance study in the Betong Division of Sarawak,^23^ which contrasted with the detection of small numbers of *P. cynomolgi* (co-infected with *P. knowlesi*) in six of 1,047 (0.6%) samples from patients with malaria at Kapit Hospital in Sarawak.^6^ Nonetheless, human co-infections with *P. cynomolgi* may still be present in Sabah at parasite densities below the detection limit of our PCR assay. Future regional surveillance for zoonotic malaria may be aided by using total nucleic acid preservation media and reverse transcription to improve the lower detection limit.^21^

The infectivity of *P. cynomolgi* in humans is greatly reduced compared with that of its natural hosts owing to its strict preferential target for human reticulocytes expressing both transferrin receptor 1 and Duffy antigen/chemokine receptor.^24^ In malariometric studies in western Cambodia using high-volume ultra-sensitive PCR, parasitemia was significantly lower in 11 individuals with asymptomatic *P. cynomolgi* infection (geometric mean 3,604 parasites/mL, upper range 26,923 parasites/mL) compared with eight individuals with asymptomatic *P. knowlesi* infection (geometric mean 52,488 parasites/mL); thus, the majority of *P. cynomolgi* infections in Cambodia were below the detection threshold of the assay used in the current study.^8^ It is also possible that interspecies competition, particularly against *P. knowlesi*, impaired *P. cynomolgi* growth within humans and the mosquito vector. Although mixed zoonotic malaria parasites are frequently detected in both infected macaques and mosquito vectors,^11^^,^^13^ *P. knowlesi* has the shortest life cycle among primate malarias, which, in addition to more efficient red blood cell invasion, may provide a growth replication advantage over *P. cynomolgi*. Of note, *Plasmodium* species co-infections may also lead to a higher parasitemia of one of the infecting species (a commensal effect), as demonstrated with *P. malariae*.^25^ Further studies are required to fully explore interactions between *P. cynomolgi* and *P. knowlesi* during the mosquito and human life cycles.

Overall, our study did not find evidence of widespread zoonotic *P. cynomolgi* transmission contributing to microscopy-positive infections in Sabah, Malaysia.
